# Cranial Ewing Sarcoma/Peripheral Primitive Neuroectodermal Tumors: A Retrospective Study Focused on Prognostic Factors and Long-Term Outcomes

**DOI:** 10.3389/fonc.2019.01023

**Published:** 2019-10-09

**Authors:** Jun Chen, Ruimin Cheng, Fanfan Fan, Yifeng Zheng, Yakun Li, Yong Chen, Yu Wang

**Affiliations:** ^1^Department of Neurosurgery, Xianning Center Hospital, Xianning, China; ^2^Department of Dermatology, Tongji Hospital, Huazhong University of Science and Technology, Wuhan, China; ^3^Department of Neurosurgery, Tongji Medical School, Tongji Hospital, Huazhong University of Science and Technology, Wuhan, China

**Keywords:** Ewing sarcoma, primitive neuroectodermal tumors, cranial, survival, prognostic factor

## Abstract

**Purpose:** Cranial Ewing sarcoma (ES)/peripheral primitive neuroectodermal tumors (pPNETs) are rarely reported because of their extremely low incidence, and the current understanding of these tumors is poor. The purpose of this study was to illustrate the clinical, radiological, and pathological features of cranial ES/pPNETs and to discuss prognostic factors by survival analysis.

**Methods:** A total of 31 patients who were pathologically diagnosed with cranial ES/pPNETs between 2000 and 2019 were enrolled in this study. To identify which parameters were associated with higher progression-free survival (PFS) and overall survival (OS) rates, univariate and multivariate analyses were performed.

**Results:** The mean follow-up period was 24.8 months (range, 1–109 months). Eighteen (58.1%) patients had local recurrence and seven (22.6%) patients had distant metastasis. The results of the univariate analysis suggest that the extent of resection and adjuvant radiotherapy are potential prognostic factors for PFS and OS. Adjuvant chemotherapy was associated with OS (*P* = 0.027) but not with PFS (*P* = 0.053). The multivariate analysis revealed that the extent of resection and adjuvant radiotherapy were independent prognostic factors for both PFS and OS. In addition, metastasis was an adverse prognostic factor for OS.

**Conclusions:** Surgical management plays a crucial role in the treatment of cranial ES/pPNETs, and gross total resection should be striven for whenever possible. Post-operative radiotherapy is highly recommended to improve PFS and OS. This study also confirms that metastasis is an adverse prognostic factor for cranial ES/pPNETs.

## Introduction

Ewing sarcoma/peripheral primitive neuroectodermal tumor (ES/pPNET) is an undifferentiated malignant, small, round cell tumor that usually arises from long bones and soft tissues in the second decade of life (1–3). It rarely occurs in the skull and meningeal tissue; only ~50 cases of cranial ES/pPNETs have been reported in the English literature (4). Given the paucity of reported cases, the clinical, radiological, and pathological features of cranial ES/pPNETs are still unclear. Therefore, cranial ES/pPNET is often misdiagnosed as an atypical teratoid/rhabdoid tumor (AT/RT) or a primary leptomeningeal medulloblastoma/cPNET, especially intracranial cPNET (5–7). In addition, because of the lack of clinical symptoms in the early stages of cranial ES/pPNET, most patients are diagnosed in advanced stages, leading to worse outcomes. Consequently, comprehensive studies on the clinical, radiological, and pathological features of cranial ES/pPNETs are warranted.

Minimal information on cranial ES/pPNET has been published due to its rarity, and most published information has come from case reports or small case series reports, in which no exact information has been reported on progression-free survival (PFS) and overall survival (OS). Our group has previously reported a series of cases to illustrate the clinical features of cranial ES/pPNETs, but the sample was too small to conduct statistical analysis (8). Therefore, it is necessary to perform a statistical analysis on a large case series to identify the prognostic factors for PFS and OS in cranial ES/pPNET. The purpose of this study was to illustrate the clinical, radiological, and pathological features of cranial ES/pPNETs and to discuss prognostic factors by survival analysis.

## Materials and Methods

A total of 31 patients with pathologically confirmed cranial ES/pPNETs were surgically treated at Tongji Hospital (Tongji Medical College, Huazhong University of Science and Technology) between February 2000 and January 2019. Medical records of all of the patients were retrospectively reviewed for clinical notes, operative details, radiographic images, and pathology reports. A brain and whole-spine magnetic resonance imaging (MRI) scan was performed in all patients before surgery. The extent of the initial surgery, the use of any post-operative adjuvant therapy, the length of follow-up, and long-term outcomes were also noted. The extent of tumor resection was recorded as total, subtotal, or partial, according to the surgical record and post-operative magnetic resonance imaging (MRI). Gross total resection (GTR) was defined as the entire lesion being resected, subtotal resection (STR) was defined as >80% of the lesion being resected, and partial resection (PR) was defined as ≤ 80% of the lesion being resected. The length of follow-up was recorded as the period from the date of initial surgery to death, or until January 2019 for living patients. The long-term outcomes we have taken in consideration are PFS and OS. The diagnosis of tumor progression, including recurrence, regrowth, and/or metastasis, was defined according to clinical manifestations and imaging results at outpatient follow-up. PFS was defined as the interval from the date of initial surgery to tumor progression or death. OS was defined as the time between initial surgery and death. The death status and date of death were obtained through telephone interviews.

## Statistical Analysis

SPSS version 20.0 (IBM Corp., Armonk, New York, USA) was applied for statistical analysis. The univariate and multivariate analyses were performed to identify independent variables that could predict prognosis. Patient factors were age, gender, and disease duration. Tumor factors were maximum tumor diameter, tumor type, Ki-67 index, brain invasion, bone invasion, and metastasis. Treatment factors were the extent of resection, adjuvant radiotherapy, and adjuvant chemotherapy. The PFS rate and OS rate were evaluated by the Kaplan–Meier method, and univariate analysis for various possible prognostic factors was performed by the log-rank test. Factors with a *P*-value ≤ 0.1 in the log-rank tests were subjected to multivariate analysis by Cox proportional hazards analysis. Statistical significance was defined as a *P*-value < 0.05.

## Results

### Clinical and Radiological Features

The clinical characteristics of the 31 patients are presented in Table 1. The population comprised 16 men and 15 women with a mean age of 19.6 (range, 1–44) years. The average duration of the initial symptoms was 40.7 (range, 1–210) days. The most common initial clinical manifestation was headache (*n* = 21; 67.7%), followed by vomiting (*n* = 14; 45.2%) and swelling over the scalp (*n* = 10; 32.3%).

**Table 1:** Patient characteristics and univariate analysis of prognostic factors affecting progression-free survival and overall survival.

**Factor**	**Number**	**Progression-free survival**		**Overall survival**	
		**Median time (months)**	***P*-value**	**Median time (months)**	***P*-value**
**AGE**					
<20/≥20 (years)	17/14	10 vs. 8	0.894	23 vs. 21	0.947
**GENDER**					
Male/female	16/15	8 vs. 8	0.651	22 vs. 21	0.977
**MAXIMUM TUMOR DIAMETER (cm)**					
≤ 5/>5	16/15	13 vs. 8	0.836	23 vs. 20	0.714
**DISEASE DURATION**					
<1/≥1 (month)	16/15	13 vs. 7	0.716	24 vs. 21	0.695
**EXTENT OF RESECTION**					
GTR/without GTR	17/14	15 vs. 6	0.007	28 vs. 13	0.008
**TUMOR TYPE**					
Solid/solid and cystic	21/10	8 vs. 13	0.914	23 vs. 21	0.696
**KI-67 INDEX**					
≤ 40/>40%	15/16	10 vs. 8	0.987	24 vs. 19	0.345
**ADJUVANT RADIOTHERAPY**					
Yes/no	18/13	14 vs. 4	0.006	25 vs. 9	0.001
**ADJUVANT CHEMOTHERAPY**					
Yes/no	19/12	13 vs. 6	0.053	23 vs. 10	0.027
**BRAIN INVASION**					
Yes/no	24/7	8 vs. 14	0.568	21 vs. 23	0.536
**BONE INVASION**					
Yes/no	12/19	8 vs. 8	0.743	23 vs. 21	0.880
**METASTASIS**					
Yes/no	7/24	–	–	14 vs. 23	0.032

*GTR, gross total resection*.

The mean tumor maximum diameter at diagnosis was 5.2 cm (range, 3–9 cm). The most common tumor location was the temporal region (*n* = 10; 32.3%), followed by the parietal region (*n* = 6; 19.4%), the frontotemporal region (*n* = 4; 12.9%), the frontal region (*n* = 3; 9.7%), the temporal-parietal region (*n* = 3; 9.7%), the occipital region (*n* = 1; 3.2%), the frontoparietal region (*n* = 1; 3.2%), the parietal-occipital region (*n* = 1; 3.2%), the tentorium supratentorial and infratentorial region (*n* = 1; 3.2%), and the left cerebellar-peduncular angle (*n* = 1; 3.2%).

**Figure 1:**
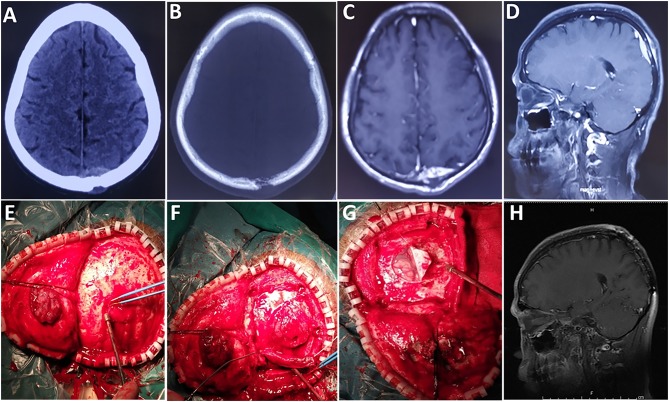
A case of epidural tumor. **(A,B)** Preoperative CT scans show that the lesion had broken through the outer table of the skull to invade the scalp. **(C)** Contrast-enhanced axial and **(D)** sagittal images show significant enhancement. **(E,F)** The tumor was reddish and soft, with an abundant blood supply. **(G)** The tumor did not invade brain tissue. **(H)** Images obtained 6 months after surgery demonstrated no local tumor recurrence.

**Figure 2:**
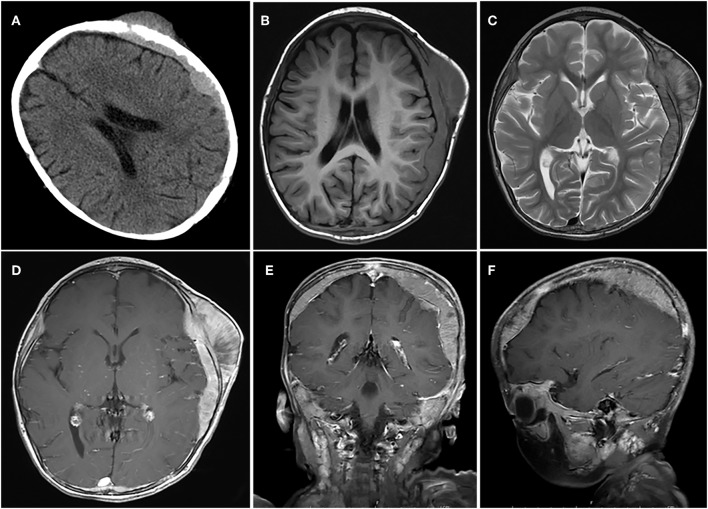
A case of huge tumor. **(A)** Axial CT scan shows show that the lesion had broken through the outer table of the skull to invade the scalp. **(B–F)** The tumor was located in the epidural and subdural space with a wide base, adjacent skull erosion, and soft tissue invasion under the scalp.

Based on computed tomography (CT) scans, 16 (51.6%) cases showed slightly high density, 12 (38.7%) cases showed isodensity, and 3 (9.7%) cases showed mixed iso–low density. According to the CT scans, bone destruction caused by tumor invasion occurred in 12 cases (Figures 1, 2).

**Figure 3:**
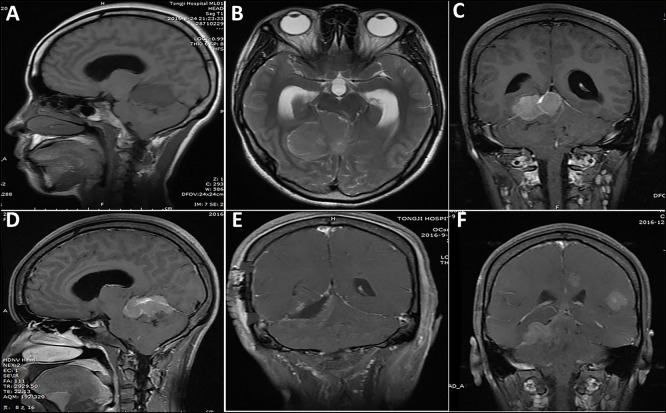
A case of lesion located in the tentorium supratentorial and infratentorial region. **(A–D)** One week before surgery, a solid appearance was observed and the border was relatively clear. **(E)** Post-operative imaging showed subtotal resection of the lesion, with a small tumor residue. **(F)** Five months after initial surgery, tumor recurrence and multiple metastasis were observed.

**Figure 4:**
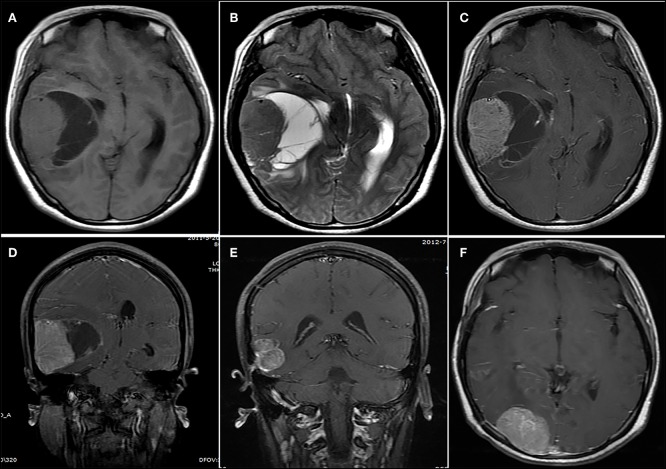
A lesion showing a concomitant cystic and solid appearance. **(A,B)** The lesion showed an isointense signal on **(A)** the T1-weighted image and **(B)** the T2-weighted image. **(C)** Contrast-enhanced axial and **(D)** coronary images show significant heterogeneous enhancement. **(E,F)** Fourteen months after initial surgery, **(E)** tumor recurrence and **(F)** metastasis were observed.

MRI images were available in all of the cases. Twenty-one (67.7%) cases showed a solid appearance (Figure 3) and 10 (32.3%) cases showed a concomitant cystic and solid appearance (Figure 4). The lesions showed hypointense T1 and hyperintense T2 signals in 15 (48.4%) cases, isointense T1 and T2 signals in 5 (16.1%) cases (Figure 4), isointense T1 and hyperintense T2 signals in 7 (22.6%) cases, and isointense T1 and mixed T2 signals in 4 (12.9%) cases. On the MRI images, the lesions showed homogeneous enhancement in 13 (41.9%) cases and heterogeneous enhancement in 18 (58.1%) cases. According to the MRI results, the lesion border was relatively well-defined (Figure 3) in 22 cases and poorly defined in nine cases.

### Pathological Features

**Figure 5:**
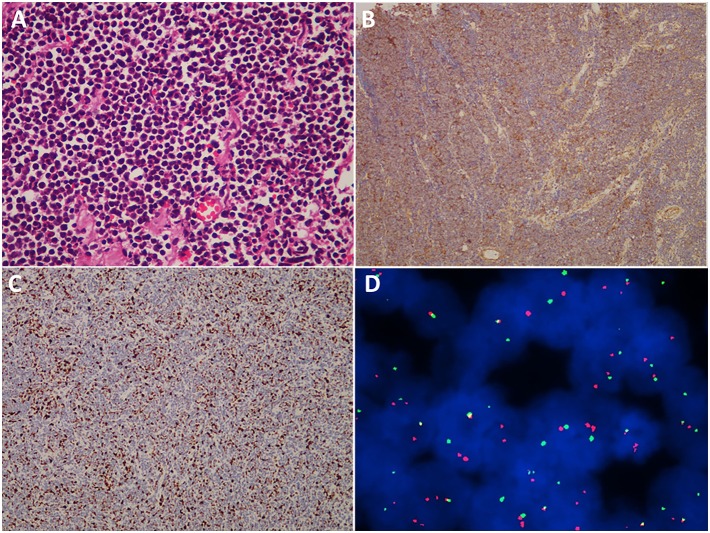
Histopathological, immunohistochemical, and cytogenetic examination of cranial ES/pPNET. **(A)** Light microscopy imaging showed a highly cellular ES/pPNET tumor consisting of uniform small, round or oval, undifferentiated cells with hyperchromatic nuclei and a scanty cytoplasmic wall (hematoxylin–eosin-stained, ×400). **(B)** Immunohistochemical staining showed positivity for CD99 (×100). **(C)** Immunohistochemistry utilizing anti-MIB-1 (Ki-67) antibodies revealed a high proliferative index (×100). **(D)** Representative FISH result using an EWSR1 (22q12) dual color break apart rearrangement probe (Vysis). ES/pPNET cells gave a yellow signal; the split pattern of one orange and one green signal is indicative of a rearrangement of one copy of the EWSR1 gene.

Light microscopic histologic examination of hematoxylin–eosin-stained slides revealed that the tumor mainly consisted of uniform small, round or oval, undifferentiated cells with hyperchromatic nuclei and a scanty cytoplasmic wall (Figure 5). Immunohistochemistry experiments revealed that 31 (100%) patients were positive for CD99 (Figure 5), 21 (67.7%) patients were positive for Vimentin, and 21 (67.8%) patients were positive for Friend Leukemia Virus Integration 1 (FLI-1). Immunohistochemistry utilizing anti-MIB-1 (Ki-67) antibodies revealed a high proliferative index in a great majority of the cases (Figure 5). The mean expression of the Ki-67 labeling index was 40% (range, 2–90%). A fluorescence *in situ* hybridization (FISH) analysis was performed in 5 cases, and EWS/FLI1 translocation was detected in 3 cases (Figure 5). However, a corresponding FISH study was not performed in the other 26 cases.

### Surgical Findings and Outcomes of Follow-up

All 31 patients underwent surgical treatment. GTR, STR, and PR were achieved in 17, 13, and 1 cases, respectively. The gross appearance of the lesions was reddish (Figure 1) or yellow. Abundant blood supply was found in 24 cases (Figure 1) and moderate blood supply in 7 cases. Fifteen cases had a pseudocapsule. Hemorrhage, necrosis, and liquefaction were found inside the tumors in 10 cases.

The mean follow-up period was 24.8 months (range, 1–109 months). Post-operative radiotherapy was performed in 18 cases, with a median dose of 50 Gy (range, 40–60 Gy). Post-operative chemotherapy was performed in 19 cases. During the follow-up, 18 (58.1%) patients had local recurrence (Figures 3, 4) and 7 (22.6%) patients had distant metastasis (Figures 3, 4). The distant metastatic sites were the intracranial metastases in five patients, the lung in one patient, and the spinal cord in one patient. The median PFS was 8.0 months. The 1-, 2-, and 5-year PFS rates were 45.1, 26.3, and 10.5%, respectively. Twenty-three patients died of tumor recurrence and/or metastasis, and the median OS was 22.0 months. The 1-, 2-, and 5-year OS rates were 74.2, 34.9, and 13.1%, respectively.

### Univariate and Multivariate Analyses of Prognostic Factors for Progression-free Survival

The results of the univariate analysis of the prognostic factors affecting PFS are presented in Table 1. Regarding the extent of resection, patients with GTR enjoyed obviously higher PFS rates compared with the other extents of resection (*P* = 0.007). Patients who underwent radiotherapy exhibited significantly higher PFS rates than those treated without radiotherapy (*P* = 0.006). There were no significant differences among the other factors (i.e., age, gender, maximum tumor diameter, disease duration, tumor type, Ki-67 index, adjuvant chemotherapy, brain invasion, and bone invasion).

**Figure 6:**
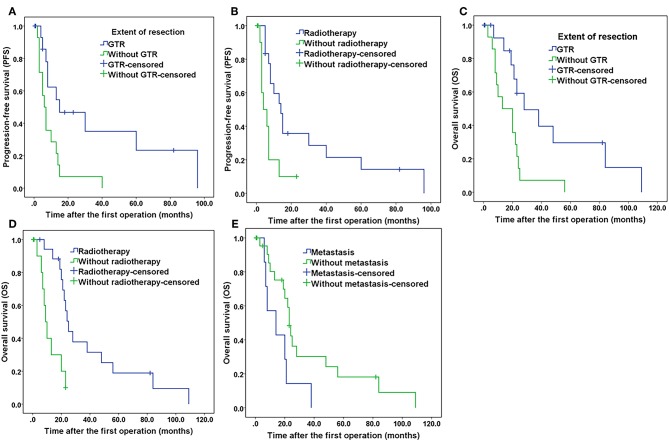
Kaplan–Meier curves of progression-free survival and overall survival. **(A)** Kaplan–Meier curves of progression-free survival for the extent of resection. **(B)** Kaplan–Meier curves of progression-free survival for patients treated with and without radiotherapy. **(C)** Kaplan–Meier curves of overall survival for the extent of resection. **(D)** Kaplan–Meier curves of overall survival of patients treated with and without radiotherapy. **(E)** Kaplan–Meier curves of overall survival for patients with and without tumor metastasis.

Potential prognostic factors, extracted by univariate analysis, were subjected to Cox proportional hazards analysis (Table 2). Multivariate analysis showed that the extent of resection (*P* = 0.026) and adjuvant radiotherapy (*P* = 0.030) were significant independent prognostic indicators. The Kaplan–Meier curves of PFS for the extent of resection and adjuvant radiotherapy are shown in Figure 6. Multivariate analysis revealed that adjuvant chemotherapy was not an independent prognostic factor for PFS. Detailed results are shown in Table 2.

**Table 2:** Multivariate analysis of prognostic factors for progression-free survival and overall survival.

**Factor**	**PFS**			**OS**		
	**HR**	**95% CI**	***P*-value**	**HR**	**95% CI**	***P*-value**
Extent of resection	2.797	1.133–6.902	0.026	4.412	1.641–11.859	0.003
Adjuvant radiotherapy	2.795	1.107–7.059	0.030	7.367	2.287–23.726	0.001
Adjuvant chemotherapy	–	–	0.783	–	–	0.577
Metastasis	–	–	–	0.179	0.061–0.528	0.002

*CI, confidence interval; HR, hazard ratio*.

### Univariate and Multivariate Analyses of Prognostic Factors for Overall Survival

The results of the univariate analysis of the possible prognostic factors affecting OS are presented in Table 1. According to our statistical analysis by the Kaplan–Meier method, a significant difference was found for the extent of resection (*P* = 0.008), adjuvant radiotherapy (*P* = 0.001), adjuvant chemotherapy (*P* = 0.027), and metastasis (*P* = 0.032). These potential prognostic factors, extracted by univariate analysis, were subjected to Cox proportional hazards analysis (Table 2). Multivariate analysis revealed that adjuvant chemotherapy (*P* = 0.577) was not an independent prognostic factor for OS. The extent of resection (*P* = 0.003), adjuvant radiotherapy (*P* = 0.001), and metastasis (*P* = 0.002) were independent prognostic factors for OS. The Kaplan–Meier curves of OS for extent of resection, adjuvant radiotherapy, and metastasis are shown in Figure 6. Additionally, details of the results of the multivariate analysis on the above four prognostic factors for OS are presented in Table 2.

## Discussion

Cranial ES/pPNET is a rare family of malignancies with high recurrence potential and poor prognosis (4). Due to its rare nature, the clinical features and prognostic factors are poorly understood. In this study, we performed univariate and multivariate analyses to investigate the clinical, radiological, and pathological prognostic factors for PFS and OS in patients with cranial ES/pPNET. The results suggest that the extent of resection and adjuvant radiotherapy were independent prognostic factors for both PFS and OS.

In our series, we found no biased gender distribution, as opposed to previous studies that reported that cranial ES/pPNET occurred with a slight male predisposition (4, 9). The patient age at disease onset was widely distributed, ranging from 1 to 44 years (mean 19.6 years), with a peak incidence in the second decade of life. This corresponded to the average age reported in most surgical case series (3, 10, 11). The most common symptoms were headache and vomiting, which were most likely due to an elevated intracranial pressure caused by the mass effect. The mean duration of symptoms before the initial surgery was 40.7 days, which is shorter than in previous reports (4). The tumors in our study were frequently located in the temporal region. Statistical analysis revealed that age, gender, and disease duration were no potential factors for the prognosis of patients (all *P* > 0.05). The same results were achieved for maximum tumor diameter, tumor type, brain invasion, and bone invasion. However, a recent study has reported that age was an independent prognostic factor for the prognosis of patients (12).

Due to histologic similarities, cranial ES/pPNET is often misdiagnosed as an AT/RT or a primary leptomeningeal medulloblastoma/cPNET, especially intracranial cPNET (5–7). Accurate diagnosis depends on immunohistochemical and molecular genetic analysis. Previous studies showed that the most sensitive and specific detection method for the diagnosis of cranial ES/pPNET was the combination of CD99 and FLI-1 immunohistochemistry (13–15). In our series, positive expression of CD99 was found in 31 (100%) cases, consistent with the diagnosis of ES/pPNET. Twenty-one (67.8%) cases were positive for FLI-1, which further confirmed the diagnosis of ES/pPNET. As is well-known, the golden standard for diagnosing ES/pPNET is the identification of the tumor type-specific fusion genes EWSR1/FLI-1 (16, 17). However, only 27 cases of cranial ES/pPNETs have been confirmed by molecular genetic analysis (3). In our study, a FISH analysis was performed in 5 cases, and EWS/FLI1 translocation was detected in three cases. In addition, our study showed that the average Ki-67 labeling index was 40%, with a range of 2–90%. Until now, studies about the connection between the Ki-67 index and PFS or OS were still lacking; in our study the Ki-67 index was not a potential prognostic factor for PFS or OS (all *P* > 0.05).

Surgical treatment is the cornerstone of therapy for cranial ES/pPNETs. The aim of surgery is to relieve symptoms, control local recurrence, achieve a sufficient volume reduction for further oncological management, and prolong patient survival. It has been reported that the extent of surgical resection is one of the most important prognostic factors for extracranial ES/pPNETs (18). In addition, some researchers reported that radical resection might result in a better prognosis for cranial ES/pPNETs than subtotal excision (4, 19). Although cranial ES/pPNET cannot be regarded as a surgically curable tumor, the role of tumor resection in patient survival cannot be overemphasized. According to the results in our study, it is of vital importance to attain GTR in the initial surgery.

Cranial ES/pPNETs are aggressive in nature and have a high tendency for local recurrence and metastasis. Other bone metastasis/focus maybe were not disclosed upfront because of the incomplete upfront work-out and thus is not certain that the cranial bone is the only focus of Ewing sarcoma for the patients. Although the ideal surgical management of malignant tumors, including cranial ES/pPNETs, consists of GTR, this may be limited by the field of view and the involvement of soft tissue. Due to the high risk of residual tumor cells and recurrence, post-operative adjuvant therapy should be conducted as soon as possible after initial surgery. Due to the paucity of reported cases, the benefit of radiotherapy and/or chemotherapy remains unclear. Some studies advocate focal radiotherapy for cranial ES/pPNETs (2, 20). Another study recommended conventional whole cranial radiotherapy after surgery (10). However, no robust direct evidence of the impact on the survival rate has been found. Consequently, it is desirable to investigate whether post-operative radiotherapy improves PFS and/or OS for patients with cranial ES/pPNETs. In our series, 18 patients received post-operative focal radiotherapy. With an intensity of 40–60 Gy could significantly improve the PFS and OS rates.

Previous studies have shown that adjuvant chemotherapy could improve the long-term survival rate of extracranial ES/pPNETs (21, 22). However, the benefit of chemotherapy in cranial ES/pPNETs has yet to be established (23). Chemotherapy is never a single treatment modality and always combines with surgery, radiotherapy, or both for local control (19). The results of our univariate analysis indicate that post-operative chemotherapy could improve OS (*P* = 0.027) but not PFS (*P* = 0.053). In our multivariate analysis, these results were not statistically significant.

We have improved diagnosis accuracy, surgical management, and post-operative adjuvant therapy, but the risk of local recurrence and distant metastasis still poses challenges for the surgeon. Post-operative recurrence is common for cranial ES/pPNETs, and 18 (58.1%) patients experienced recurrence in our study. Recurrence may increase the difficulty of reoperation or result in tumor progression and ultimately death. In addition, seven patients, including three who underwent GTR, experienced distant metastasis. Finally, our findings reveal that metastasis was an adverse prognostic factor for cranial ES/pPNETs.

To our knowledge, this is the largest series of cranial ES/pPNET cases from a single neurosurgical center, with the longest follow-up duration. All 31 patients came from a single institution, and thus, our study population is relatively homogeneous. Nevertheless, there are some limitations. Firstly, this study is a retrospective review of a rare disease, and thus, the risk of statistical bias exists. Secondly, we only focused on surgical cases, and we neglected patients who did not receive surgical treatment. Thirdly, due to the short follow-up duration, the OS rate may appear higher than it is in reality.

## Conclusions

Cranial ES/pPNET is a challenging clinical entity due to its high local recurrence rate. Surgical management plays a crucial role in the treatment of cranial ES/pPNETs, and GTR should be striven for whenever possible. Post-operative radiotherapy is highly recommended to improve PFS and OS rates. This study also confirms that metastasis is an adverse prognostic factor for cranial ES/pPNETs.

## Data Availability Statement

All datasets generated for this study are included in the manuscript/supplementary files.

## Ethics Statement

This study was a retrospective study and did not involve any experimental interventions, according to the rules of the ethics committee of Tongji Hospital, it did not require special ethics approval.

## Author Contributions

YW and JC: study design. JC, FF, RC, YZ, and YL: data collections. JC and YC: data analysis. JC: writing. All authors reviewed the manuscript.

### Conflict of Interest

The authors declare that the research was conducted in the absence of any commercial or financial relationships that could be construed as a potential conflict of interest.
